# Insights into temporal patterns of hospital patient safety from routinely collected electronic data

**DOI:** 10.1186/2047-2501-3-S1-S2

**Published:** 2015-02-24

**Authors:** Blanca Gallego, Farah Magrabi, Oscar Perez Concha, Ying Wang, Enrico Coiera

**Affiliations:** 1 Centre for Health Informatics, Australian Institute of Health Innovation, University of New South Wales, Kensington NSW 2052, Australia

**Keywords:** Patient safety, Electronic health records, Health services research

## Abstract

**Background:**

The last two decades have seen an unprecedented growth in initiatives aimed to improve patient safety. For the most part, however, evidence of their impact remains controversial. At the same time, the healthcare industry has experienced an also unprecedented growth in the amount and variety of available electronic data.

**Methods:**

In this paper, we provide a review of the use of routinely collected electronic data in the identification, analysis and surveillance of temporal patterns of patient safety.

**Results:**

Two important temporal patterns of the safety of hospitalised patients were identified and discussed: long-term trends related to changes in clinical practice and healthcare policy; and shorter term patterns related to variations in workforce and resources. We found that consistency in reporting is intrinsically related to availability of large-scale, fit-for-purpose data. Consistent reported trends of patient harms included an increase in the incidence of post-operative sepsis and a decrease in central-line associated bloodstream infections. Improvement in the treatment of specific diseases, such as cardiac conditions, has also been demonstrated. Linkage of hospital data with other datasets provides essential temporal information about errors, as well as information about unsuspected system deficiencies. It has played an important role in the measurement and analysis of the effects of off-hours hospital operation.

**Conclusions:**

Measuring temporal patterns of patient safety is still inadequate with electronic health records not yet playing an important role. Patient safety interventions should not be implemented without a strategy for continuous monitoring of their effect.

## Introduction

Administrators, healthcare providers and researchers struggle to decide which practices are most cost-effective in the improvement of patient safety. A major reason behind this problem is the fragmented and specialised nature of modern healthcare systems. This fragmentation occurs at different levels, among in-patient, out-patient and general practice care as well as among hospital wards. This results in poor information flows, disjointed care and inadequate boundaries of legal and financial responsibilities, all of which negatively influence quality of care and patient safety, and make it harder to understand and measure it [1,2]. Furthermore, hospitals, like any other organisation, need to adapt to changes in their environment, such as ageing population, more sophisticated technologies, temporal disease patterns, variations in workforce and changes in funding [3]. Appropriate continuous measurement of temporal patterns of patient safety in and beyond the hospital has become essential to inform hospital patient safety strategies.

With the growing availability of electronic routinely collected data inside hospitals and across the healthcare system, we have the opportunity to track patient safety over time and explore its temporal 'rhythms'. One of the criticisms of the use of routinely collected data for measuring quality of care is the fact that data may not be fit for purpose. Many measure gaps have been identified e.g. related to patient functional status, patient at the end of life, continuity of care, and some important process measures. Nevertheless, the continuous growth of the adoption of electronic health records (EHRs), new ways of sharing data via distributed data networks, together with better standardisation will slowly make the task of measuring patient safety easier.

In this paper we provide a review of the role of administrative datasets (including linked data), claims datasets, registries, surveillance systems and EHRs, in the detection and analysis of temporal patterns in hospital patient safety. We identified two important aspects in the temporal dynamics of healthcare delivery in hospitals, which are intrinsically linked to patient safety: (a) Sustained changes in processes of care and patient outcomes following the adoption of new technologies or new policies; these changes are often slow, taking place on the order of months or years. (b) Temporal variations related to workforce and resource patterns including weekend and after-hours hospital operation, hospital shifts and yearly influx of new graduates; this dynamics encompasses hourly, daily or monthly changes. A summary of our findings is displayed in Table 1.

**Table 1:** Summary of patient safety related temporal patterns measured by routinely collected data.

	Observations	Datasets	Examples
**Long term trends**: Effect of changes in clinical practice and healthcare policy	Temporal trends in adverse events	• Hospital administrative data (mostly using PSI) • Registries (e.g. cardiac-related) • National Surveillance systems (e.g. nosocomial infections) • Pharmacy and clinical laboratory data • Electronic Health Records	• Sustained decrease in central line-associated bloodstream infections [62,63,65] • Sustained increase in post-operative sepsis [51-55] and post-operative thromboembolism [51-53]
			
	Temporal trends in performance measures		• Sustained improvement in treatment and outcomes of cardiac conditions [34,58,59,61,67]
			
	Changes in process measures and patient outcomes associated with patient-safety interventions		• Surgical safety checklist for reduction of surgical AEs[68]. • Subglottic secretion drainage for the prevention of ventilator-associated pneumonia [69]. • Sterile barriers and antibiotic-impregnated catheters for the reduction of catheter-related blood stream infections [66]

**Hourly, weekly and monthly variations**: Effect of changes in workforce and resources	Weekend and after-hours effect	• Hospital administrative data • Registries (e.g. death, cardiac surgery)	• Increased in-hospital mortality for weekend admissions among some patient groups 77 • Increased 7 day post-admission (in-hospital and post-discharge) mortality for weekend admissions for some patient groups [80] • Weekend and after-hours AMI admissions less likely to receive timely cardiac procedures [81,83] • Only 5% of six urgent procedures were performed on the weekend [87]
			
	July effect		• Increase in mortality and decrease in efficiency after new residents after influx of junior residents [96] • Increased LOS and mortality rates in teaching hospitals related to residency turnover [97] • Significant spike in fatal medication errors during July [98]

### Measuring hospital patient safety using routinely collected electronic data

The oldest and most widely-used measure of patient safety is mortality. Without appropriate context, however, this important patient outcome is a crude measure of safety since it does not provide a complete picture. Indeed, the use of hospital standardised mortality ratios as a measure of quality of care has been heavily criticised [4,5]. Part of the problem stems from our inability to predict when death was preventable and, in particular, when patients are at the end of their natural life [4,6]. Nevertheless, for specific subpopulations and in conjunction with other relevant information, mortality remains an important outcome measure of safety. Other outcomes of care that can be easily extracted from administrative datasets, and that have been correlated with patient harm are hospital length of stay [7] and unplanned hospital readmissions, particularly in relation to drug adverse events [8]. Linkage of hospital administrative data with death registries, emergency department visits data, public and private claims data, and other administrative databases have proved useful to measure mortality post-discharge, re-admissions, or history of previous hospitalisations.

The patient outcome measures most closely associated to patient safety are potentially preventable adverse events, as defined by the Institute of Medicine [9]. Traditionally, the gold standard for reporting these patient harms is through manual review of medical records by expert clinicians (see review [10]). This is a labour intensive job that requires at least two independent evaluators, who often present low to moderate rates of agreement. In order to facilitate this task, special methods to systematically review medical records to screen for 'triggers' of potential patient harm have been developed. The most commonly used is the Global Trigger Tool [11].

A more cost-effective method of detecting patient harm events can be achieved using routinely electronically captured information. Automated screening using administrative and claim records searches for adverse events among diagnosis and procedural codes. For example, the Medicare Patient Safety Monitoring System [12] is a US surveillance system that uses administrative and inpatient Medicare discharges to screen for adverse events. Medical records of the selected patients are then reviewed by experts, reducing the cost per chart reviewed. One drawback of this system is the potential for missing adverse events. The Agency for Healthcare Research and Quality also developed a set of Patient Safety Indicators (PSI) to recognise patient safety incidents from administrative data [13,14]. The limitations of administrative data, in general, and of the PSIs in particular, have been pointed out in several studies [15]. Mostly, administrative coding was generated for the purpose of reimbursement, and therefore lacks clinical content and context. Some adverse events are poorly represented by PSIs, which have been found to have positive predictive values (PPV) ranging from 28% for postoperative hip fracture to 87% for postoperative wound dehiscence [16]. Nevertheless, the creation of newer codes (e.g. [17]) and, in particular, of present-on-admission (POA) flags (to discriminate between pre-existing and hospital-acquired conditions) [18,19] have increased the validity of these measures.

Some of the limitations encountered when using administrative data alone can be reduced when combined with other electronic datasets, such as pharmacy and clinical laboratory data. These additional sources of information increase the knowledge of illness severity and can signal specific adverse events such as sudden adverse drug reactions [20,21]. Another useful source of routinely collected information easily linked to administrative data and now also implemented in electronic format is discharge summaries. The clinical narratives of discharge summaries have proven useful in increasing the specificity of adverse event detection tools [22,23]. Because a significant percentage of patient safety events take place post-discharge [24] or in an outpatient setting [25], linking datasets across types of care are needed to measure the full extent of the effect of hospital adverse events.

Eventually, information integrated in EHRs will provide the ideal source for automated detection of patient safety events. The use of EHRs allows better identification of clinically relevant patient groups [20,21,26,27] and has the potential to greatly improve the cost-effectiveness of audit processes [21]. Also, it provides a way to analyse the health care system as a whole and not as independent health care units. Already, automated identification of drug adverse events and hospital-acquired infections has been implemented using information technology (see e.g. [28]). Furthermore, in contrast to administrative and claims data, EHRs are recorded in near real time allowing for earlier surveillance of patient safety [29]. Although levels of adoption of EHRs are still low [30,31], and the quality of EHRs today is highly variable [32], as the secondary use of EHRs becomes more prevalent, and appropriate standards are introduced and validated, we expect availability and quality of EHR data to improve.

National surveillance systems and registries offer high-quality long-term counts of specific patient safety events. One of the oldest running surveillance systems of hospital adverse events is the Centers for Disease Control and Prevention's National Nosocomial Infections Surveillance (NNIS) system [33] (now National Healthcare Safety Network), established in 1970. Data is collected using standardised protocols and it is classified by hospital ward, associated clinical procedure or device, and infection type using information from pathology. Registries were designed to collect patient-level, fit-for-purpose, data around specific interventions or conditions in order to be able to compare institutions, devices/procedures and patients, as well as to monitor temporal trends. The most ubiquitous registries relate to cardiac surgery and cardiac interventions [34].

Incident reporting systems, where hospital personnel voluntarily and confidentially report incidents, represent another repository of patient safety events. Data from established large incident reporting systems usually contain a structured taxonomy that classifies the patient safety event together with free narrative describing the event [35]. Although this data represents only a very small (under 5%) and biased sample (mainly identification, falls and medication errors) of actual patient safety events, it provides complementary information about near misses and the clinical context and contributing factors leading to patient harm [36,37]. Studies of incidents reported by patients after hospitalisation suggest that patient reports are generally reliable [38,39]. However, preferred ways of soliciting patient information still involve resource-consuming methods such as in-person or phone interviews. Adding questions regarding adverse events to current electronic hospital patient surveys, or extending existing incident report systems to patients are some of the cost-effective ways to build these important consumer databases. It is also possible to query and analyse electronic datasets of medical-malpractice claims. This data also represents a very small and biased fraction of all the adverse events related to medical negligence [37,40].

Due to limitations in measuring patient harms, process of care measures have been included as additional indicators of patient safety, particularly in the context of evaluation of hospital performance [41]. Process measures should have a demonstrable causal relation with multiple patient outcomes. Examples of evidence-based process measures are antibiotic prophylaxis before surgery [42] and aspirin at arrival for patients with acute myocardial infarction [43]. Most processes of care measures are only captured in medical records or registries.

The analysis of large sets of routine clinical data plays an important role in post-marketing surveillance of harmful or ineffective treatment plans. Many adverse events associated with drugs or devices are too infrequent to be detected in randomised clinical trials, or may affect patient populations with specific co-morbidities, often excluded from experimental studies. Observational studies using large routine clinical datasets can also generate hypotheses, which can guide clinical trial design, thus providing a safer and more efficient way of generating medical evidence.

### Temporal trends: the effect of changes in clinical practice and healthcare policy

Little has been said regarding the sustained widespread effects on patient safety that arise from changes in clinical practice and healthcare policy. Observational studies designed to analyse the effect of safety interventions often report short-term changes in local settings and are sometimes controversial [44-47]. Reasons for this poor surveillance include the need to balance sustained improvement versus the possibility of improvement trends unrelated to the intervention, lack of good quality data that is fit for purpose, and the complexity in evaluating patient safety practice [48,49]. On the other hand, studies analysing observed long-term, large-scale temporal trends in processes of care and patient outcomes cannot easily unravel the contribution of specific interventions; and temporal electronic data of detailed adoption and implementation of new clinical practices and policies is generally lacking. An additional difficulty arises from the need to account for changes in recording and reporting methods over time.

The temporal evolution in the incidence of hospital patient harm events has been measured using review of medical records with the Global Trigger Tool (GTT) [50]; PSIs [51-55] and diagnostic codes [56] from administrative datasets; disease-specific performance measures and selected outcomes collected from registries [57,58] and national surveillance programs [59-63] and malpractice claims [64].

**Figure 1:**
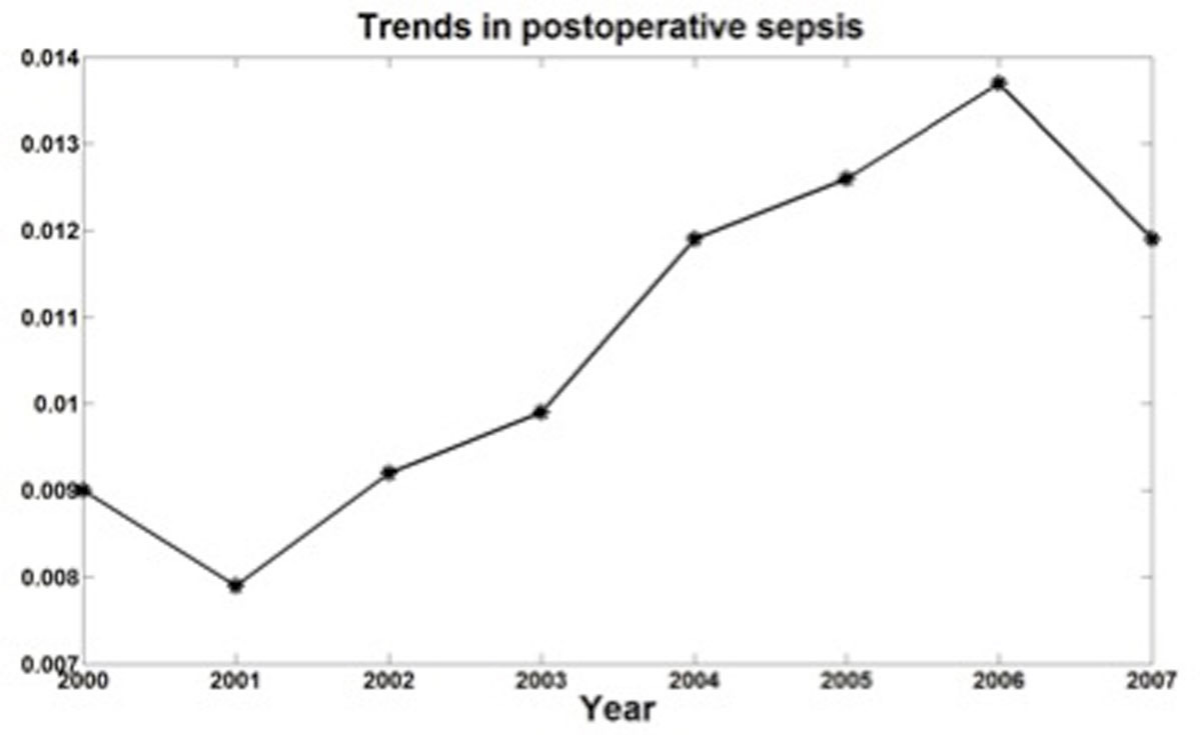
**Trends in postoperative sepsis**. Rates of post-operative sepsis (PSI 13 as defined by AHRQ: Surgical discharges age 18 and older with a diagnostic code (ICD10-AM) of sepsis in any secondary diagnosis field/Surgical discharges age 18 and older) in 501 hospitals in New South Wales, Australia from July 2000 till July 2007.

While Landrigan et al found no evidence of widespread improvement between 2002 and 2007 in 10 North Carolina hospitals [50]; studies using PSIs and larger inpatient datasets found significant (albeit sometimes differing) temporal trends. For example, studies of approximately 1,000 US hospitals found a decreased trend of iatrogenic pneumothorax between 1995 and 2000 [51] and between 1998 and 2007 [53]. On the other hand, study [52] described an increased trend on the same patient harm in 108 Veteran's Health hospitals from 2001 to 2004. Post-operative sepsis and post-operative venous thromboembolism events were found to increase in all above studies. The increase in the rate of post-operative sepsis has been confirmed by other studies [54,55]. This has also been found by the authors of this review among hospitals in New South Wales (NSW), Australia (see Figure 1).

Reasons behind these observed changes in the rates of specific adverse events are not usually properly elucidated and discussed in the literature. It is believed that sustained changes are achieved via multistep, multilevel efforts involving clinicians, regulatory agencies and hospital administrators alike. A good example of a successful sustained reduction in patient harm is the prevention of bloodstream infections. Trends in central line-associated bloodstream infections in the US show a marked decrease since the late 90 s [62,63,65]. Pronovost and colleagues [66] describe the effort behind this success as a combination of measurement, dissemination and mobilisation across many stakeholders. Evidence on the history of the evolution of safety in hospitals also indicates that the greatest safety gains often come from the introduction and subsequent widespread adoption of safer drugs and procedures.

Analyses of performance indicators for selected conditions have found consistent improvement among a large random sample of inpatient datasets in the US in 2001 as compared to 1998 [60]; as well as across 3,000 accredited US hospitals in 2004 as compared to 2002 [61]. In particular, treatment of cardiac conditions, such as acute myocardial infarction, has undergone numerous well-documented changes in the last couple of decades, which have significantly improved the safety of patients suffering from these conditions [34,58,59,61,67]. Improvement was observed in relation to the adoption of new techniques (e.g. use of coronary stent instead of atherectomy devices) and evidence-based recommendations (e.g. rapid administration of thrombolytic therapy), as well as in patient outcomes (e.g. length of stay, death).

There is no lack of studies relating specific safety interventions to improvements in the reduction of harm. A discussion on evidence-based patient safety practices can be found at [44]. The best understood patient safety practices with higher strength of evidence relate to focus interventions aimed at reducing specific patient harms. Examples of successful interventions include the surgical safety checklist [68], which has been linked to a significant reduction in death and complications associated with non-cardiac surgery across the world; the use of subglottic secretion drainage for the prevention of ventilator-associated pneumonia [69]; and the use of sterile barriers and antibiotic-impregnated catheters for the reduction of catheter-related blood stream infections [66]. Analyses of the impact of more general patient safety interventions are often controversial or inconclusive. For example, some of the more recent, across the board, quality of care interventions in the US: pay for performance [70,71], restriction of specific procedures to centers of excellence [56,72], and work hour regulations [47,73], lack positive conclusive evidence.

Examinations of trends in patient safety require the use of large, fit-for-purpose data, such as that extracted from longitudinal medical records. Available temporal trends show a mixed picture with improvement in the treatment of some conditions and worsening or no-change in others. There have been extensive efforts on improving patient safety across the world. However, due to limitations in measures and data sources, the effects of most of these efforts remain unclear.

### Hourly, weekly and monthly variations: the effect of changes in workforce and resources

When patients go to hospital, they expect to receive high levels of safety and care at all times. However, using routinely collected electronic data, several studies have demonstrated the existence of higher risk of death and adverse events at specific times of the day, days of the week, and months of the year. These periods of decreased safety have been related to both changes in staff numbers and composition, as well as lack of access to specialised clinical facilities.

It is well known that hospitals during weekdays between approximately 8 am and 6 pm and hospitals during off-hours (weekends and after-hours) are two very different clinical environments [74]. Off-hours hospitals have only a small percentage of administrative and clinical teams, with no senior managers, almost no consultants or specialists and significantly lower nurse-to-patient ratios. These differences in staff composition and numbers are known to worry both junior doctors as well as hospital chief executive officers. Hospital residents perceive excessive work hours, inadequate supervision and problems with handoffs as the most common reasons for mistakes [75]. Similarly, nurses perceive personal neglect, heavy workload and changes in staff as the main factors in medication errors [76]. The relationship between staff numbers, particularly nurse staffing, and patient outcomes has also been documented (see e.g. [77]). On the other hand, access to some clinical facilities, such as diagnostic testing and specialised theaters, is also often not available outside normal operating hours. This reality has made researchers hypothesise a lower quality of care during weekends and after-hours, particularly for conditions that require complex immediate care outside emergency departments and intensive care units [78].

**Figure 2:**
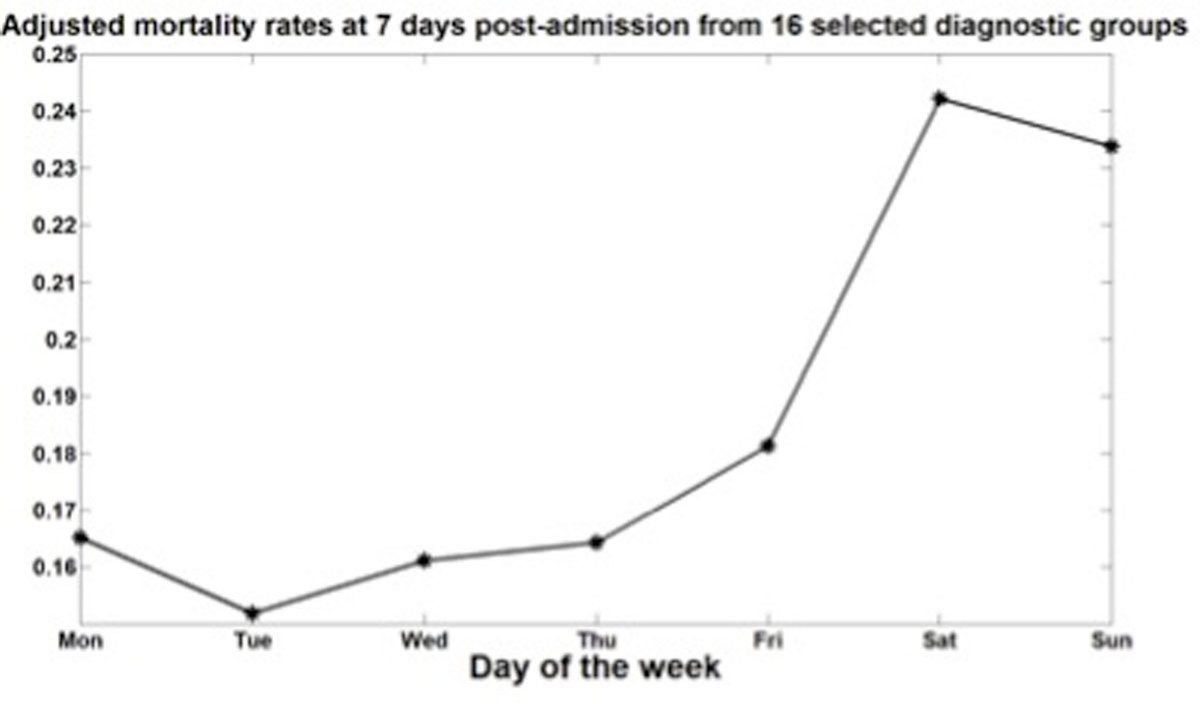
**Adjusted mortality rates at 7 days post-admission from 16 selected diagnostic groups**. Mortality rates at 7 days post-admission for 16 selected diagnostic groups within emergency admissions to 501 hospitals in New South Wales, Australia between July 2000 and July 2007. Mortality rates were adjusted by sex, age, and Charlson morbidity index. Diagnostic groups were selected as those for which there was a statistically significant 'weekend effect' (see [80]).

Using large hospital administrative datasets, researchers have found that mortality among some patient groups admitted on weekends via emergency departments is higher than for those patients admitted on weekdays (see Figure 2); and that there are no groups for which weekend admission is safer [79,80]. This phenomenon has also been demonstrated in studies focusing on special conditions (see e.g. [78,81-84]). In some of these studies, linkage with mortality registries allowed for the analysis of deaths post-discharge [80-84]. In order to help discriminate between lower quality of care and sicker patients presenting to emergency departments in the weekend, Perez-Concha et al looked at the survival curves associated with various patient groups during one week post-admission; they found that for some patients (e.g. patients with heart conditions), failing to provide immediate care during the weekend appeared to be the reason behind the increased risk in mortality, while for others (e.g. cancer patients) the observed 'weekend effect' was likely to be due to differences in patient condition [80]. Linking births and deaths certificates has shown increase in the risk of neonatal death after-hours [85,86]. Other outcomes such as length of stay [87], and re-admissions [81] have also been observed to increase for emergency weekend admissions. Errors in ordering medications during the overnight period have also found to increase, particularly by postgraduate year 1 doctors [88].

When looking at the 'weekend effect' more closely, researchers have found that there are measurable differences in patient treatments. In particular, it has been observed that the incidence and timing of important interventional procedures differs for weekend vs. weekday patients. For example, off-hours patients presenting with acute myocardial infarction are less likely to receive immediate cardiac procedures [81,83]; and experience substantially longer door-to-balloon times [89]. Indeed, Kostis et al [83] found that statistically significant differences in 30 days mortality became nonsignificant after adjusting for invasive cardiac procedures. Similarly Bell et al [87] analysed time to six prespecified procedures among more than 100,000 emergency admissions in Canada, and found that only 5% of urgent procedures were performed on the weekend. Delays in interventional procedures have been independently related to infection complications, length of stay and mortality [90,91].

The effect of weekends and evenings has also been found when comparing the outcomes from elective surgery among days of the week [92,93]; for patients suffering from an in-hospital cardiac arrest [94]and for adults admitted to and discharged from intensive care [95].

Another phenomenon observed using routinely collected data is the relationship between patient outcomes and the cyclic rotation of trainee doctors, which takes place in teaching hospitals. In the Northern Hemisphere the influx of new or junior residents occurs in July, and therefore the existence of this period of observed decreased patient safety has been coined the 'July effect'. Researchers hypothesise that this staff turnover disrupts established doctor teams abruptly decreasing the average experience of the workforce. Young and colleagues performed a review of the literature regarding this phenomenon in 2011 [96]. They concluded that, in general, existing studies demonstrate an increase in mortality and a decrease in efficiency (reported as length of stay, duration of procedure and hospital charges) related to the end of the year changeovers. The 'July effect', like the 'weekend effect', does not affect all patient groups and a substantial degree of heterogeneity among studies has been found; however, its evidence is generally weaker. One of the largest studies of the effect of cohort turnover involved roughly 20% of all US hospitals for a period of 5 years [97]. By comparing temporal trends of teaching vs non-teaching hospitals, the authors found increased average length of stay and mortality rates related to residency turnover. Another large study including over 60 million death certificates and more than 200,000 medication errors, found a significant spike in fatal medication errors at medical institutions during July [98].

Evidence of the impact of differences in standard of care, such as out-of-hours care, could not have been revealed without the use of large routinely collected electronic data. More informative data and further research are needed to unravel the causes behind these periods of decreased safety and provide appropriate solutions.

## Discussion

In this paper we provide a review of the current use of routinely collected electronic data to identify and analyse temporal patterns of hospital patient safety. We found that the science of measuring patient safety is still not properly advanced and surrounded by controversy. The gold standard of counting patient harms is still manual review of medical records, and use of full EHRs for this purpose is far from widespread. This is an important problem, since it is well known that trust in measures and the measurement process facilitate the use of the recorded information for training and improvement. For those situations in which there is suitable large-scale temporal data, it is much easier to find consistent trends. One example is the observed decrease in central line-associated bloodstream infections in the US, captured by the national surveillance program on nosocomial infections.

Assessing the impact of patient safety interventions is currently poorly done. Most studies only report pre and post intervention effects in local settings without any further monitoring of sustained change. Often these reports are controversial. We propose that interventions should always be implemented together with a strategy for continuous monitoring of the target patient outcome.

Data linkage has proved useful in the unravelling of important temporal aspects of patient safety; for example, death and birth registries, data containing the day and time of interventional procedures and clinical datasets that help to better determine the illness severity of patients. However, some useful patient-safety related datasets, such as information about staffing numbers in each hospital shift and information about locum personnel, are mostly unavailable. A notable exception is the case of some hospitals in Massachusetts which provide voluntary unit-by-unit reports on caregiver staffing levels updated on an annual basis for over 750 hospital units including emergency departments [99].

Some of the temporal patterns discussed in this review warrant further investigation. A worrisome increase in the incidence of post-operative sepsis, for example, has been found in several studies. New evidence on the negative effect of weekend and after-hours hospital operation has made researchers argue about what should be the most cost-effective way of improving off-hours hospital care [81]. If the cause of the 'weekend effect' is understaffing then the strategy should be to regulate workforce. If, however, the cause is inappropriate treatment (such as delays to surgery) not related to staffing numbers then a more cost-effective strategy could be rewarding good off-hours performance. Information technology can also help reducing the gap between the 'normal' and the 'off-hours' hospital by creating safety nets and providing information for junior doctors when senior staff are not present.

Analyses of routinely collected electronic records provide essential insights into temporal patterns of hospital patient safety. They reveal the existence of non-clinical temporal patterns related to hospital workflow and allow for the monitoring of the effect of patient safety interventions. However, there are still important information gaps with respect to both patients (clinical and social status) as well as hospitals (staffing numbers, availability of specialised facilities). The use of integrated EHRs will be important to fill in some of these gaps and provide more meaningful measures of safety.

## Funding and ethics statement

This work was funded by National Health and Medical Research Council (NHMRC) Program Grant 568612, and Project Grant 1045548; and approved by the NSW Population and Health Services Research Ethics Committee and the UNSW Human Research Ethics Committee. Its contents are the responsibility of the authors and their institutions and do not reflect the views of the NHMRC.

## Competing interests

The authors declare that they have no competing interests.

## Declarations

Publication costs were funded by NHMRC Program Grant 568612

This article has been published as part of *Health Information Science and Systems *Volume 3 Supplement 1, 2015: Proceedings of the Health Informatics Society of Australia Big Data Conference (HISA 2013). The full contents of the supplement are available online at http://www.hissjournal.com/supplements/3/S1/
